# Porcelain Facings

**Published:** 1915-11

**Authors:** John H. Penhale, Harold E. Rice


### PORCELAIN FACINGS.

READ BEFORE THE STUDENTS' DENTAL SOCIETY OF THE UNIVERSITY OF MICHIGAN, MAY 17, 1915.

BY JOHN H. PENHALE AND HAROLD E. RICE.

Everyone is more or less familiar with the long pin facing and some of its uses in crown and bridge work. We have found out that it is liable to check during soldering, due to our negligence of a few essential points. These facings are made of two materials, a mineral and a metal, each of which possesses physical properties which are affected differently by the heat to which they are subjected in soldering. This trouble from unequal expansion and contraction may be overcome by slowly and uniformly heating the piece to be soldered, giving the facing and its metal pin a chance to expand slowly and equally, thus saving the checking due to this cause. The adaptation of the backing which allows edges of metal to overlap the porcelain facing will cause it to check, due to the shrinkage of the solder in cooling. Sometimes the holes in the metal backing are much too large for the pins, allowing the solder to run under the backing and so check the facing on cooling. Carelessness in bending the pins to retain the backing, preparatory to soldering will cause an uneven strain at the point of their insertion in the porcelain. The method of investment may be at fault and if the technique, perfect in every other detail, a mistake in the material or its use will bring about the fatal check, and an otherwise beautiful piece of work will be ruined.

It is always a good method to paint the porcelain with whiting, being careful not to paint where you wish solder to flow. A solution of whiting in alcohol dries quickly and much time is saved if this little precaution saves the facing. If the investment chips out a little close to the facing and a little flux run on to it, a check is likely to be the result. The whiting saves this. Another point which aids in soldering a Richmond crown, is when it is ready to invest, be sure to have a little excess of wax on the buccal side at the cervical joint between the backing of the facing and the cap. This, when melted out, leaves a little space for the solder which runs in and closes the joint perfectly. It is much easier to grind a little off than to reinvest and solder over. These facings are much used in individual crowns and in bridge work where a crown of strength is required. The esthetic is often sacrificed for strength, however, as necessarily gold must show to a greater or lesser extent, depending on the skill of the operator.

The not infrequent presentation of broken facings after a piece of work is set permanently, combined with the more or less difficult operation of replacing them in a secure and artistic manner, has resulted in the introduction of several varieties of detachable and replaceable facings, which are applicable to the construction of dowel crowns as well as bridge work, though perhaps more generally to the latter.

When a facing has successfully gone through the fire it may be fractured after being set permanently; fracture in this case may be due to one of three reasons; either a faulty adaptation of the backing wherein it affords insufficient strength; inadequate protection of the occlusal edges; or a total disregard of the requirements of occlusion. The use of a style which is easily replaceable has doubtless advantage in some instances, as the faulty backing is often combined with severe strain of the porcelain, due to some condition of malocclusion, or the patient sometimes subjecting the piece to very rough usage. Almost any practical means of facilitating repair in the event of accident is decidedly useful. The advantages claimed by the advocates of replaceable facings are: first, that the porcelain is not subjected to variations of heat in soldering; second, facings may be more easily replaced in the event of their becoming fractured; third, the probability of becoming fractured from usage is greatly diminished, because the facing is not rigidly attached to the metal backing; fourth, the color is not changed.

The first point made is practically the weakest, because the fracturing of a facing during the process of soldering it to any kind of an attachment is inexcusable, and can be almost invariably attributed to either lack of skill, or a neglect of detail. The second must be regarded as problematical, at least in the manner in which these facings are ordinarily used, for the reason that it is often impossible to properly adapt any style of facing to the individual case without considerable grinding. In such cases the replacement of an exact duplicate of the same mould would require much skill in regrinding a new facing to refit the case. The third feature presents the most practical and plausible advantage, because a porcelain facing supported by mechanical means, supplemented with an intervening medium such as gutta percha or cement, which affords a somewhat cushion-like effect, will withstand greater stress than one held firm and rigid. Hence fracture is less likely to occur since the facing will yield slightly to stress before breaking. The fourth point of advantage is doubtful, for the reason that any change of color is usually due to excessive heat, or the presence of a discoloring backing, as has been previously mentioned.

It has been suggested that when preparing these facings, for work, two of them be prepared for each piece, and the second one kept in a box with the patient's name, so in case a breakage occurs, the same can be inserted with little trouble.

Of the detachable facings, we have Dwight's, Mason's, Roach's and Steele's. The last is the one used to the greatest extent at this time. These include both anterior and posterior facings, and they are more applicable in bridgework than for individual crowns. The Goslee crown is used in this connection to some extent and the Davis crown may also be ground in, soldered, or used with a cast base. The ideal tooth is the one which presents as much porcelain as possible, this porcelain not being weakened by the presence of metal pins, nor by the provisions for retention, and which therefore possesses a maximum strength and which will require but a minimum grinding in effecting the desired and required adaptation. The Goslee interchangeable crown and bridge tooth meets these requirements. These teeth, including the Davis crown and Steele facing are all made in numerous moulds and colors, so that there should be no difficulty in finding a tooth to fit the case. In the use of the Goslee tooth, using a soldered under base, the root is first prepared for the crown, the plate of 36 or 38 gauge 24K is either burnished directly to the tooth, or made to fit the base of the crown by the indirect method. The Goslee facing is then fitted as closely as possible to the case. A piece of pure gold is then burnished to under surface of facing and the facing set upon the tooth, ready for soldering. A Davis crown post is set into the plate on a root and soldered, another Davis crown post is cut off at the head and soldered to the plate which has been burnished to the facing. The whole is invested now and soldered between the two plates of gold. In the cast under process, the base is made of inlay wax, the Davis post heated and forced down into root canal through the wax, and the facing set on in place and surplus wax trimmed as desired, after which it is removed, invested, and cast in 24K gold. Another way is to burnish a piece of 24K gold to the root set in the pin and solder, after which the face of the crown is carved up as before, in inlay wax, invested and cast to plate and dowel. The same detail is also applicable in the use of the Steele's facing, except that each facing has its individual backing ready made to fit and all that is necessary is to burnish, remove facing, and either solder or cast to backing.

